# QTL for Yield Traits and Their Association with Functional Genes in Response to Phosphorus Deficiency in *Brassica napus*

**DOI:** 10.1371/journal.pone.0054559

**Published:** 2013-01-28

**Authors:** Taoxiong Shi, Ruiyuan Li, Zunkang Zhao, Guangda Ding, Yan Long, Jinling Meng, Fangsen Xu, Lei Shi

**Affiliations:** 1 National Key Laboratory of Crop Genetic Improvement and National Centre of Plant Gene Research, Huazhong Agricultural University, Wuhan, China; 2 Key Laboratory of Arable Land Conservation (Middle and Lower Reaches of Yangtze River), Ministry of Agriculture, Huazhong Agricultural University, Wuhan, China; USDA-ARS, United States of America

## Abstract

**Background:**

Oilseed rape (*Brassica napus* L.) is one of the most important oil crops. A primary limitation to the cultivation of this crop is the lack of available phosphorus (P) in soils. To elucidate the genetic control of P deficiency tolerance in *Brassica napus*, quantitative trait locus (QTL) for seed yield and yield related-traits in response to P deficiency were identified using a double haploid mapping population (TN DH) derived from a cross between a P-efficient cultivar, Ningyou 7 and a P-inefficient cultivar, Tapidor.

**Results:**

Three field trials were conducted to determine seed yield (SY), plant height (PH), number of primary branches (BN), height to the first primary branch (FBH), relative first primary branch height (RBH), pod number per plant (PN), seed number per pod (SN) and seed weight of 1,000 seeds (SW) in 188 lines of TN DH population exposed to low P (LP) and optimal P (OP) conditions. P deficiency decreased PH, BN, SN, PN and SY, and increased FBH and RBH with no effect on SW. Three reproducible LP-specific QTL regions were identified on chromosomes A2, A3 and A5 that controlled SN, PN and SW respectively. In addition, six reproducible constitutive regions were also mapped with two each for SY-LP on A2, and FBH-LP on C6 and one each for PH-LP and SW-LP on A3. About 30 markers derived from 19 orthologous genes involved in *Arabidopsis* P homeostasis were mapped on 24 QTL regions by comparative mapping between *Arabidopsis* and *Brassica napus*. Among these genes, *GPT1*, *MGD2* and *SIZ1* were associated with two major loci regulating SY-LP and other yield-related traits on A2 between 77.1 and 95.0 cM.

**Conclusion:**

The stable QTLs detected under LP conditions and their candidate genes may provide useful information for marker-assisted selection in breeding high-yield *B. napus* varieties with improved P efficiency.

## Introduction

Phosphorus (P) is one of the essential macronutrients for plants and is a major limitation for plant development worldwide due to its low availability and inaccessibility in soil [1]–[3]. To mitigate this, P fertilizers are applied to improve P availability in soils. However, cost and low recovery rates of P fertilizers not only increase the cost of crop production but also lead to serious environmental pollution and exhaust non-renewable phosphate resources. Therefore, a sustainable strategy for crop production would be to breed crops with high efficiency in acquiring P from native soil reserves or fertilizer sources.

Nutrient-efficient plants produce higher yields per unit of nutrient applied or absorbed compared with other plants grown under similar agroecological conditions [4]. One way to determine the genetic basis of tolerance to nutrient deficiency is to map quantitative trait loci (QTL) based on molecular markers. A number of QTLs associated with P efficiency traits have been detected in rice [5]–[7], maize [8], wheat [9], [10], common bean [11], [12] and soybean [13], [14]. Using a soybean *F_9_* recombinant inbred lines (RILs), 13 QTLs associated with root traits and 18 associated with P-efficiency were identified under low P and high P conditions, and three QTL clusters were found to be associated with traits for root and P-efficiency at low P levels [14]. In wheat, each QTL detected for P use efficiency (PUE) was found to be linked to QTLs regulating agronomic traits [10]. *Pup1* is a major locus that confers P deficiency tolerance in rice [5], [7], [15], [16]. Overexpression of a *Pup1*-specific protein kinase gene (*PSTOL1*) in phosphorus-starvation-intolerant varieties significantly enhanced grain yield in phosphorus-deficient soil [17]. These results provide strong evidence for the hypothesis that enhancing P efficiency would improve agronomic performance of crops.

*Brassica napus* is one of the most important oilseed crops worldwide and is extremely sensitive to P availability in soil. Since high seed yield is one of the desired targets in *B. napus* breeding, various studies have reported the importance of identifying P-efficient germplasm in *B. napus* [18]. QTLs for seed yield related-traits in response to P deficiency were first identified in *B. napus* using an *F_10_* RIL population in two crop seasons. In these experiments, a number of low-P-specific QTLs were detected across seasons. Four markers developed from genes involved in *Arabidopsis* P homeostasis were mapped to the confidence intervals of QTLs associated with a P efficiency coefficient (the ratio of seed yield at low P level to that at adequate P level) or low-P-specific QTLs [19]. Identification of QTLs associated with root traits and P-efficiency parameters partially revealed the genetic basis of tolerance to P deficiency and also confirmed the importance of root morphology traits in adaption to low P levels [20], [21]. High colinearity and synteny between the *Brassica* species and *Arabidopsis* genomes have allowed *in silico* mapping of multiple *Arabidopsis thaliana* genes involved in P metabolic pathway as well as genes controlling agronomical traits in *B. napus* QTLs. These include QTLs for shoot mineral concentrations [22], seed mineral concentrations [23], root morphological traits [20], [21], flowering time [24] and seed yield traits [19], [25]. The sequence of *B. rapa* genome [26] and *B. oleracea* genomes (http://www.ocri-genomics.org/) has further facilitated identification of conserved genome segments and prediction of homologous genes for target traits in *B. napus*, in addition to confirming QTLs detected previously for shoot P content and P uptake efficiency related traits in *Brassica* species [21]–[23], [27]–[30].

In the present study, a double haploid (DH) population derived from a cross between P-efficient *cv.* Ningyou 7 and P-inefficient *cv.* Tapidor (TN DH population) was used to determine seed yield and yield-related traits under low P (LP) and optimal P (OP) conditions in three trials conducted during four years. In addition, QTLs controlling seed yield and yield-related traits conferring P-deficiency tolerance were also determined. Comparative mapping between the linkage groups of TN DH map (*B. napus*), and the *B. rapa* and *B*. *oleracea* genomes was also performed to associate QTLs with orthologous genes involved in tolerance to P deficiency. QTLs that confer P efficiency and their candidate genes would provide useful information to better understand P efficiency and to develop molecular markers to improve seed yield in *B. napus* cultivated in environments with low P.

## Results

### Phenotypic analysis of the tested traits in TN DH population

**Figure 1:**
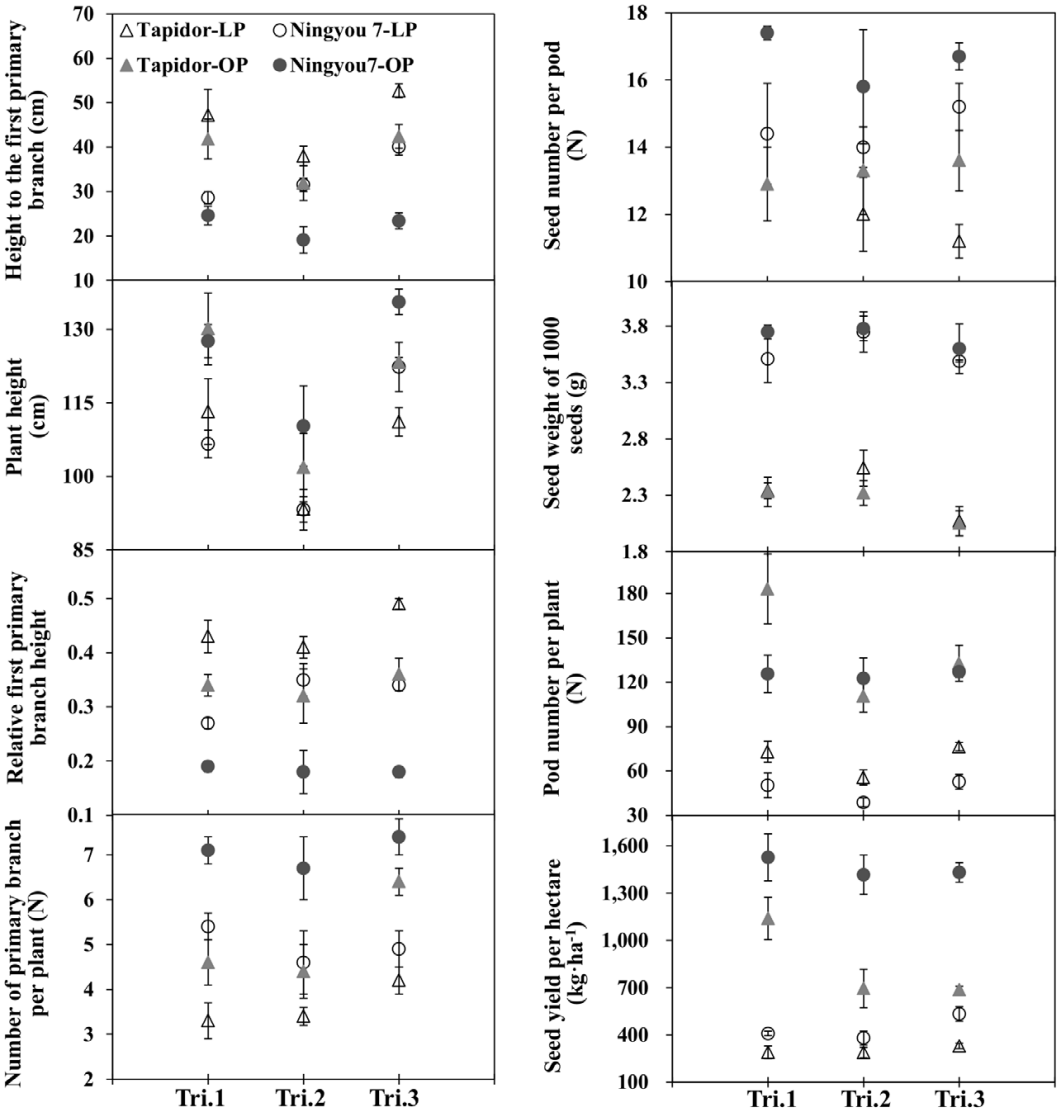
Trait differences between Tapidor and Ningyou 7 under low (LP) and optimal phosphorus (OP) conditions in three field trials. Field trial conducted from Sept. 2008 to May 2009 (Tri.1), field trial conducted from Sept. 2009 to May 2010 (Tri.2), field trial conducted from Sept. 2010 to May 2011 (Tri.3).

Phenotypic differences in the tested traits between the two parental lines under low P (LP) and optimal P (OP) conditions are presented in Figure 1. Compared to the P-inefficient parent ‘Tapidor’, the P-efficient parent ‘Ningyou 7’ had higher number of primary branches (BN), seed weight of 1,000 seeds (SW), seed number per pod (SN) and seed yield (SY), and lower height to the first primary branch (FBH) and relative first primary branch height (the ratio of FBH to plant height (PH), RBH) under both P conditions. However, average pod number per plant (PN) in Ningyou 7 was lower than Tapidor under LP conditions. There was no significant difference in PH between the TN DH parents under two P conditions in Tri.1 (field trial conducted from Sept. 2008 to May 2009) and Tri.2 (field trial conducted from Sept. 2009 to May 2010), while PH in Ningyou 7 was higher than Tapidor in Tri.3 (field trial conducted from Sept. 2010 to May 2011). Each parent experienced lower PH, BN, SN, PN and SY in P deficient than optimal P conditions. In contrast, each parent had greater FBH and RBH in low than optimal conditions and experienced no different in SW across P levels.

**Figure 2:**
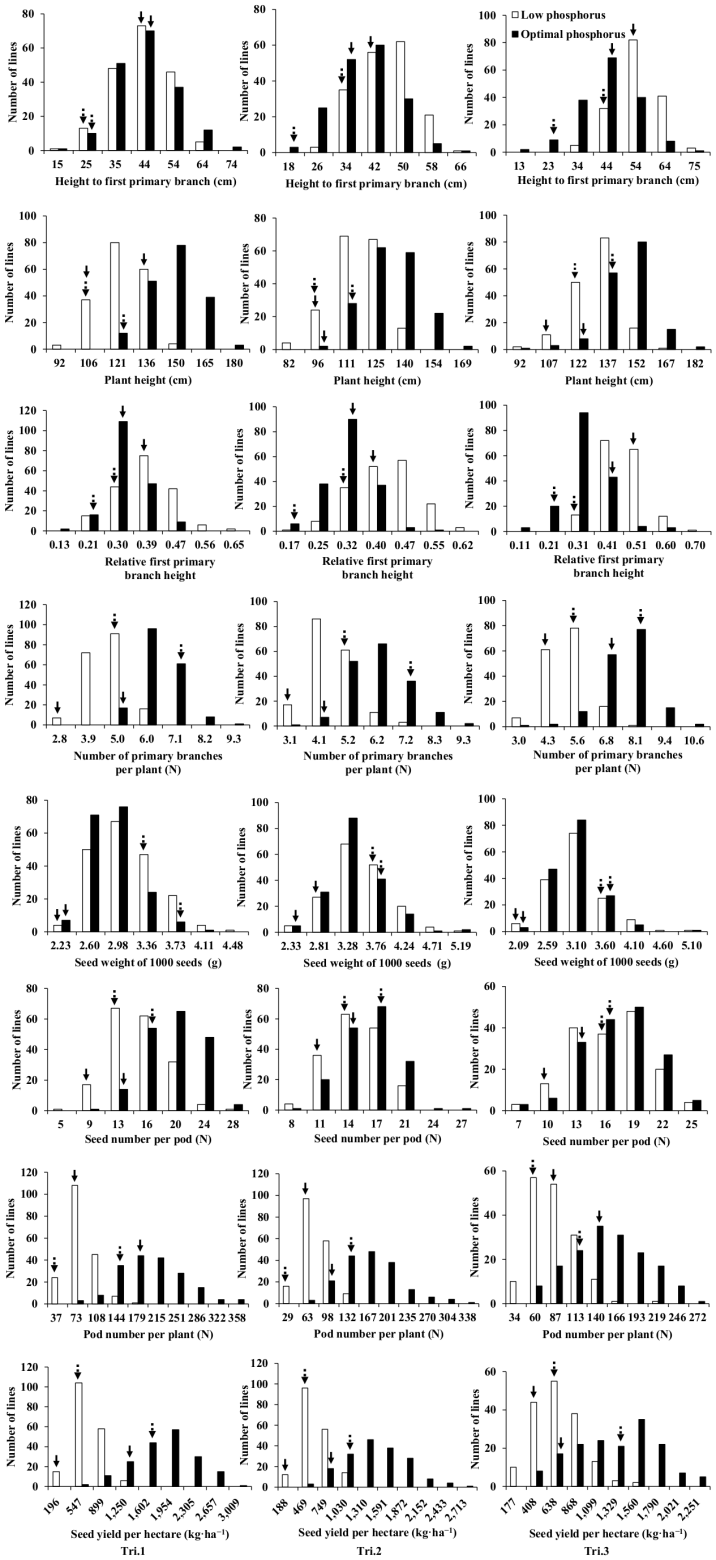
Frequency distributions of the seed yield and yield-related traits in a double haploid mapping population (TN DH) derived from a cross between a P-efficient cultivar, Ningyou 7 and a P-inefficient cultivar, Tapidor under low and optimal phosphorus conditions in three field trials. Field trial conducted from Sept. 2008 to May 2009 (Tri.1; column of graphs on the left), field trial conducted from Sept. 2009 to May 2010 (Tri.2; column of graphs on the middle), field trial conducted from Sept. 2010 to May 2011 (Tri.3; column of graphs on the right). Solid arrows indicate ‘Tapidor’, and dashed arrows indicate ‘Ningyou 7’.

TN DH population exposed to P deficiency also showed lower PH, BN, SN, PN and SY, and higher FBH and RBH with no effect on SW (Table 1 and Table S1; Figure 2). The most significant influence of P deficiency was on PN (decreasing by 67.2% in Tri.1, 61.6% in Tri.2 and 48.9% in Tri.3) followed by BN (decreasing by 32.2% in Tri.1, 28.1% in Tri.2 and 34.8% in Tri.3) and RBH (increasing by 21.4% in Tri.1, 39.3% in Tri.2 and 46.4% Tri.3). Genotypic variation in each tested trait also existed in TN DH population under both P conditions. Moreover, variation coefficients for BN, SN, PN and SY in LP conditions were larger than in OP conditions in all three trials (Table 1). In addition to the strong effects of environment, genotype, environment×P level and genotype×P level interactions for most traits, there were also strong effects of P level on all traits (Table 2). Transgressive segregation and continuous distribution was observed for each trait in TN DH population exposed to both P conditions in all three trials (Figure 2).

**Table 1:** Trait differences in a double haploid mapping population (TN DH) derived from a cross between a P-efficient cultivar, Ningyou 7 and a P-inefficient cultivar, Tapidor under low (LP) and optimal phosphorus (OP) conditions in three field trials.

Trait	P level	Tri.1			Tri.2			Tri.3		
		mean	range	cv%	mean	range	cv%	mean	range	cv%
FBH	LP	38.3	13.7–61.2	23.5	41.2	19.2–59.9	19.6	49.2	28.9–69.4	15.6
	OP	38.5	8.0–66.0	25.5	39.0	10.0–69.0	25.0	35.0	14.0–62.3	24.3
PH	LP	116.0	84.5–143.3	9.6	108.2	74.5–137.2	10.9	124.1	84.0–153.6	9.5
	OP	140.7	106.8–172.5	9.0	124.6	89.0–161.6	11.0	138.2	84.0–174.5	9.1
RBH	LP	0.34	0.15–0.61	24.7	0.39	0.17–0.59	21.1	0.41	0.23–0.66	16.9
	OP	0.28	0.09–0.47	21.2	0.28	0.14–0.48	20.8	0.28	0.07–0.55	27.1
BN	LP	4.0	2.3–6.0	17.0	4.1	2.6–7.0	19.4	4.5	2.5–7.5	19.2
	OP	5.9	4.4–8.8	12.5	5.7	2.8–8.8	18.3	6.9	2.5–10.0	16.2
SW	LP	2.88	2.04–4.18	14.1	3.25	2.10–4.91	15.1	2.83	1.86–4.77	16.2
	OP	2.93	2.10–4.30	13.9	3.16	2.09–4.95	14.6	2.78	1.84–4.85	15.0
SN	LP	13.3	3.0–25.3	28.4	13.5	6.3–20.3	21.6	14.8	5.3–22.9	26.6
	OP	17.8	7.9–26.2	19.9	14.9	7.3–25.5	20.5	15.5	5.7–23.5	23.0
PN	LP	61.4	19.0–148.5	40.2	57.9	12.0–111.5	37.8	71.3	20.7–142.3	37.3
	OP	187.0	43.0–340.0	29.9	150.6	33.0–321.0	34.3	139.6	35.5–259.1	34.8
SY	LP	464.1	19.7–1155.6	44.2	434.9	48.0–1007.3	42.4	539.0	61.9–1450.0	49.7
	OP	1649.7	455.7–2832.8	28.2	1271.0	326.7–2572.9	33.0	1163.9	239.5–2135.7	39.2

Note: Height to the first primary branch (cm; FBH), plant height (cm; PH), relative first primary branch height (the ratio of FBH to PH; RBH), number of primary branches per plant (N; BN), seed weight of 1,000 seeds (g per 1000 seeds; SW), seed number per pod (N; SN), pod number per plant (N; PN), seed yield per hectare (kg·ha^−1^; SY). Field trial conducted from Sept. 2008 to May 2009 (Tri.1), field trial conducted from Sept. 2009 to May 2010 (Tri.2), field trial conducted from Sept. 2010 to May 2011 (Tri.3).

**Table 2:** Significance of three-way ANOVA analysis for the eight traits among a double haploid mapping population (TN DH) derived from a cross between a P-efficient cultivar, Ningyou 7 and a P-inefficient cultivar, Tapidor under low and optimal phosphorus conditions in three field trials.

Source		FBH	PH	RBH	BN	SW	SN	PN	SY
Genotype	S.S	152331.1	262362.9	8.8	1029.7	396.1	16713.8	1260597.2	3915.2
	d.f.s	187	187	187	187	187	187	187	187
	significant	***	***	***	***	***	***	***	***
P level	S.S	19382.3	200903.8	5.60	2838.0	0.4	2403.1	3861317.7	9484.5
	d.f.s	1	1	1	1	1	1	1	1
	significant	***	***	***	***	**	***	***	***
Environment	S.S	12902.1	109287.7	0.4	415.1	67.2	918.1	141888.4	591.8
	d.f.s	2	2	2	2	2	2	2	2
	significant	***	***	***	***	***	***	***	***
Genotype×P level	S.S	9690.8	35805.5	1.3	230.0	25.6	2357.8	590475.3	1075.8
	d.f.s	186	186	186	186	187	187	186	187
	significant	***	***	***	**	***	***	***	***
Environment×P level	S.S	15061.4	16992.1	0.4	77.3	2.4	1375.3	233904.2	789.2
	d.f.s	2	2	2	2	2	2	2	2
	significant	***	***	***	***	***	***	***	***
Environment×Genotype	S.S	38349.0	67629.7	2.7	584.0	103.6	4389.6	712967.7	1094.2
	d.f.s	342	341	343	343	350	344	345	345
	significant	***	***	***	***	***	***	***	***

Note: Height to the first primary branch (cm; FBH), plant height (cm; PH), relative first primary branch height (the ratio of FBH to PH; RBH), number of primary branches per plant (N; BN), seed weight of 1,000 seeds (g per 1000 seeds; SW), seed number per pod (N; SN), pod number per plant (N; PN), seed yield per hectare (kg·ha^−1^; SY). Sums of squares (S.S), degrees of freedom (d.f.s).

*** *P*<0.001,

** *P*<0.01.

Pearson's correlation coefficient between traits was also calculated (Table 3). The phenotypic correlation between SY and yield-related traits under LP was similar to OP conditions in the TN DH population. SY was positively correlated to PH, BN, SN and PN, and negatively correlated to RBH. However, higher correlation coefficients between SY and PH, RBH, SN, and lower correlation coefficients between SY and PN were observed under LP conditions than under OP conditions (Table 3).

**Table 3:** Pearson's correlation coefficients among traits in a double haploid mapping population (TN DH) derived from a cross between a P-efficient cultivar, Ningyou 7 and a P-inefficient cultivar, Tapidor under low (below diagonal) and optimal phosphorus (above diagonal) conditions.

	FBH	PH	RBH	BN	SW	SN	PN	SY
FBH		0.52**	0.89**	−0.22**	−0.25**	ns	ns	ns
PH	0.45**		0.15*	0.25**	ns	0.19*	0.16*	0.22**
RBH	0.74**	ns		−0.38**	−0.23**	ns	−0.21**	−0.22**
BN	−0.22**	0.26**	−0.36**		0.01	ns	0.36**	0.39**
SW	−0.16*	−0.08	ns	ns		ns	−0.19*	ns
SN	ns	0.28**	ns	0.20*	−0.05		ns	0.52**
PN	ns	0.22**	−0.21**	0.30**	−0.19*	0.24**		0.70**
SY	ns	0.31**	−0.33**	0.39**	ns	0.58**	0.55**	

Note: Height to the first primary branch (cm; FBH), plant height (cm; PH), relative first primary branch height (the ratio of FBH to PH; RBH), number of primary branches per plant (N; BN), seed weight of 1,000 seeds (g per 1000 seeds; SW), seed number per pod (N; SN), pod number per plant (N; PN), seed yield per hectare (kg·ha^−1^; SY).

** *P*<0.01,

* *P*<0.05.

### QTL for seed yield and yield-related traits

**Figure 3:**
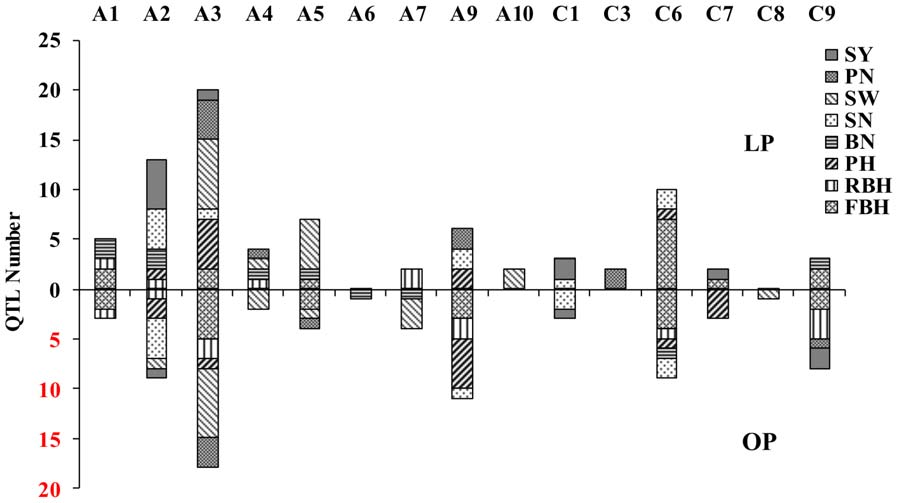
Distribution of quantitative trait loci (QTLs) associated with seed yield and yield-related traits on fifteen linkage groups under low (LP; above x-axis) and optimal phosphorus (OP; below x-axis) conditions in three field trials. Height to the first primary branch (cm; FBH), plant height (cm; PH), relative first primary branch height (the ratio of FBH to PH; RBH), number of primary branches per plant (No; BN), seed weight of 1,000 seeds (g per 1000 seeds; SW), seed number per pod (N; SN), pod number per plant (N; PN), seed yield per hectare (kg·ha^−1^; SY).

**Figure 4:**
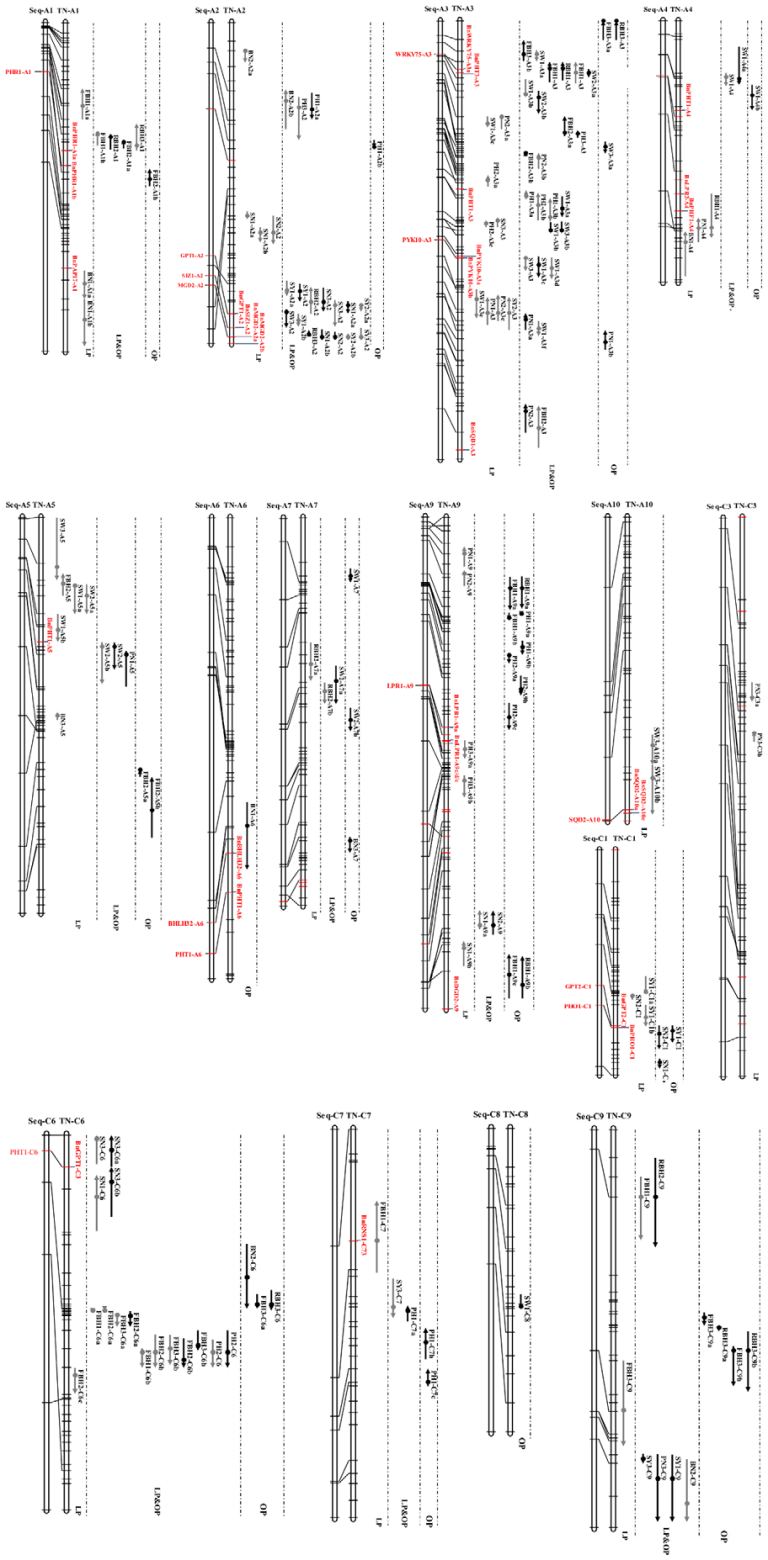
Location of quantitative trait loci (QTLs) on the linkage groups of *B. napus* and the genes aligned with *B.rapa* and *B.oleracea*. Hollow bars on the left represent the genome of *B. rapa* and *B. olerecea*, and horizontal-crossing lines indicate gene positions. Hollow bars on the right represent the linkage groups of *B.napus* and horizontal-crossing lines indicate the position of molecular markers. To the right of the linkage groups, the vertical lines with arrows indicate the QTL, with the length of each line indicating the confidence interval and circles indicating the peak position. Upward arrows represent alleles from Tapidor, and downward arrows represent alleles from Ningyou 7. Gray and black bars represent QTL detected under low (LP) and optimal phosphorus (OP) condition, respectively. Height to the first primary branch (cm; FBH), plant height (cm; PH), relative first primary branch height (the ratio of FBH to PH; RBH), number of primary branches per plant (N; BN), seed weight of 1,000 seeds (g per 1000 seeds; SW), seed number per pod (N; SN), pod number per plant (N; PN), seed yield per hectare (kg·ha^−1^; SY). Trait name and the related trial number are labeled near each QTL line. Markers shown in red and oriented on the right of the *B. napus* hollow bars represent gene-based markers (GBM) mapped in QTL intervals in *B.napus* and the corresponding homologous genes aligned to *B.rapa* and *B.oleracea* are showed in the left.

A total of 155 significant QTLs were identified for the eight tested traits from all three trials, including 79 QTLs detected under LP conditions and 76 under OP conditions (Figure 3 and Table S2). Intervals of several QTLs overlapped with each other resulting in clustering of the 155 QTLs into 81 regions on 15 chromosomes. A majority (80.0%) of these regions was distributed on A2, A3, A5, A9, C6 and C9 (Figure 3). Of the 81 QTL regions, 29 were specifically detected under LP condition, and 29 specifically under OP condition. The remaining 23 QTL regions were detected under both P conditions (Figure 4 and Table S2).

The 29 LP-specific regions were derived from 42 QTLs detected under LP conditions, with individual phenotypic contributions of QTL (*R^2^*) ranging from 6.1 to 12.9% (Figure 4 and Table S2). Three of the 29 LP-specific regions were consistently associated with the same trait in two of the three trials and mapped between 56.4–66.4 cM on A2, 80.0–92.1 cM on A3 and 0.0–28.2 cM on A5 and controlled SN, PN and SW respectively. The consistency of PN locus on A3 also coincided with QTLs for SY-LP and SW-LP. Furthermore, there were four LP-specific regions where QTLs for different traits were also observed. For example, *qPN-LP2-A3a* and *qSW-LP1-A3c* were co-located between 27.8–32.3 cM on A3; *qSN-LP3-A3* and *qPH-LP2-A3c* were observed between 58.5–61.5 cM on A3; *qRBH-LP1-A4*, *qPN-LP3-A4* and *qBN-LP1-A4* were clustered between 57.5–84.9 cM on A4; and *qSY-LP1-C1a* was associated with *qSN-LP2-C1* located between 40.8–48.6 cM on C1.

The 29 OP-specific QTL regions were integrated from 40 QTLs detected under OP conditions, with individual *R^2^* ranging from 5.9–17.6% (Figure 4 and Table S2). Among the 29 OP-specific regions, two regions were consistently mapped between 59.8–63.4 cM on A3 and 36.9–43.7 cM on A9 that regulate SW and PH respectively in two of the three field trials. In region 56.9–64.5 cM on C1, *qSY-OP1-C1* coincided with *qSN-OP2-C1* and the QTLs for FBH and RBH were co-located in five regions: 0.0–5.7 cM on A3, 18.2–28.1 cM and 128.2–141.2 cM on A9, 28.9–46.4 cM on C6 and 54.1–70.3 cM on C9. In addition, QTL for FBH also overlapped with QTL for PH in two regions: 28.4–34.8 cM on A3 and 28.7–31.0 cM on A9.

The 23 constitutive QTL regions detected under both P conditions were integrated from 73 QTLs, with individual *R^2^* varying from 5.7 to 20.2% (Figure 4 and Table S2). Six regions were considered as major loci that respond to LP deficiency because these QTLs were consistently associated with the same trait under LP conditions in at least two trials. QTLs for SY-LP were detected in two of the three trials and mapped in the 77.1–91.3 cM and 91.3–95.0 cM regions on A2 accounting for 10.8–13.0% and 8.4–14.2% of SY-LP variation, respectively, and both also coincided with QTLs for SN-OP. The reproducible locus for PH-LP was located between 50.2–59.9 cM on A3, accounting for 7.9–9.2% of PH-LP variation. The reproducible locus for SW-LP was located in 70.2–77.0 cM region on A3, explaining 7.8–11.5% of SW-LP variation. The two major regions affecting FBH-LP were detected in all three field trials and mapped in the intervals of 46.0–50.8 cM and 51.6–61.5 cM on C6, explaining 9.2–17.8% and 12.4–18.5% of FBH-LP variation respectively.

### Association of QTLs with functional genes involved in P homeostasis

In total, 30 gene-based markers (GBMs) associated with 19 P homeostasis genes in *Arabidopsis* were found to be located in the QTL confidence intervals. These 30 GBMs were grouped into six functional categories according to the recent functional classification of the corresponding *Arabidopsis* genes (Table 4). P transporter category was the largest group with 10 GBMs. Eleven GBM markers including BnPAP17-A1, BnLPR2-A4, BnPHF1-A4, BnPHT1-A5, BnLPR1-A9c/d/e, BnSQD2-A10a/c, BnGPT2-C1 and BnRNS1-C7 derived from eight genes were associated with LP-specific QTL regions. Eight GBM markers (BnPHR1-A1b, BnPHT1-A3/A4/A6, BnBHLH32-A6, BnLPR1-A9a, BnDGD2-A9 and BnPHO1-C1) derived from six genes were related to OP-specific QTL regions. Eleven GBM markers (BnPHR1-A1a, BnGPT1-A2, BnSIZ1-A2, BnMGD2-A2a/b, BnWRKY75-A3, BnPHT3-A3, BnPYK10-A3a/b, BnSQD1-A3 and BnPHT1-C6) derived from nine genes were located in the constitutive QTL regions.

**Table 4:** Gene-based markers (GBM) associated with Quantitative trait loci (QTLs) regulating seed yield-related traits and their functions in *Arabidopsis.*

GBM	Chr.	QTL region	Trait	Gene name	Function in *Arabidopsis*
*P assimilation*					
BnPAP17-A1	A1	74.0–82.5	BN-LP	*PAP17*	Encodes acid phosphatase
BnRNS1-C7	C7	19.1–38.5	FBH-LP	*RNS1*	Encodes ribonuclease
*Metal binding*					
BnLPR2-A4	A4	57.5–84.9	RBH-LP	*LPR2*	Controlling low Pi–triggered root growth inhibition
BnLPR1-A9c/d/e	A9	66.0–71.5	PH-LP	*LPR1*	Controlling low Pi–triggered root growth inhibition
BnLPR1-A9a	A9	55.1–63.1	PH-OP	*LPR1*	Controlling low Pi–triggered root growth inhibition
*P transporter*					
BnPHF1-A4	A4	57.5–84.9	RBH-LP	*PHF1*	Enables the endoplasmic reticulum exit of a high-affinity phosphate transporter
BnPHT1-A5	A5	30.9–37.7	SW-LP	*PHT1;4*	Encodes phosphate (Pi) transporters
BnPHT1-A3	A3	28.4–34.8	FBH-OP, FBH-LP, RBH-OP, SW-OP	*PHT1;4*	Encodes phosphate (Pi) transporters
BnPHT1-A4	A4	21.4–29.7	SW-OP	*PHT1;8*	Encodes phosphate (Pi) transporters
BnPHT1-C6	C6	0.0–8.4	SN-LP, SN-OP	*PHT1*	Encodes phosphate (Pi) transporters
BnPHT3-A3	A3	12.2–18.6	FBH-LP, FBH-OP, RBH-OP, SW-OP	*PHT3;1*	Transmembrane phosphate transporter
BnGPT1-A2	A2	77.1–91.3	SW-OP, SY-LP	*GPT1*	Glucose-6-phosphate transporter
BnGPT2-C1	C1	50.2–57.2	SY-LP	*GPT2*	Glucose-6-phosphate transporter
BnPHO1-C1	C1	56.9–64.5	SY-OP, SN-OP	*PHO1*	Xylem loading of inorganic phosphate
*Phospholipid metabolism*					
BnSQD1-A3	A3	114.0–127.3	PN-OP	*SQD1*	Sulfotransferase activity
BnSQD2-A10a/c	A10	72.8–87.3	SW-LP	*SQD2*	Sulfotransferase activity
BnMGD2-A2a/b	A2	91.3–95.0	RBH-OP, SN-OP,SY-LP	*MGD2*	Encodes Type-B monogalactosyldiacylglycerol synthases
BnDGD2-A9	A9	128.2–141.2	FBH-OP, RBH-OP	*DGD2*	Encodes a UDP-galactose-dependent digalactosyldiacylglycerol synthase
*Carbohydrate metabolism*					
BnPYK10-A3a/b	A3	70.2–77.0	SW-LP, SW-OP	*PYK10*	Encodes beta-glucosidase, hydrolyzing O-glycosyl compounds
*Transcriptional factor*					
BnSIZ1-A2	A2	77.1–91.3	RBH-OP, SN-OP,SY-LP	*SIZ1*	SUMO ligase activity, a focal controller of Pi starvation-dependent response
BnPHR1-A1a	A1	30.1–38.2	FBH-LP, FBH-OP, RBH-OP, RBH-LP	*PHR1*	MYB transcriptional activator of Pi starvation-responsive genes
BnPHR1-A1b	A1	43.6–48.8	FBH-OP	*PHR1*	MYB transcriptional activator of Pi starvation-responsive genes
BnBHLH32-A6	A6	93.1–102.8	BN-OP	*BHLH32*	Cellular response to phosphate starvation
BnPHT1-A6	A6	93.1–102.8	BN-OP	*PHT1;8*	Encodes phosphate (Pi) transporters
BnWRKY75-A3	A3	12.2–18.6	FBH-LP, FBH-OP, RBH-OP,SW-OP	*WRKY75*	Transcription factor modulates *PHT1* expression

Note: Height to the first primary branch (cm; FBH), plant height (cm; PH), relative first primary branch height (the ratio of FBH to PH; RBH), number of primary branches per plant (N; BN), seed weight of 1,000 seeds (g per 1000 seeds; SW), seed number per pod (N; SN), pod number per plant (N; PN), seed yield per hectare (kg•ha^−1^; SY), low phosphorus (LP), optimal phosphorus (OP).

Twelve of the 19 genes distributed in the QTL confidence intervals were mapped to corresponding genetic positions on *B. rapa* or *B. oleracea* genomes included genes related to P transport (*PHT1;8*, *PHO1*, *GPT1* and *GPT2*), transcriptional control (*SIZ*, *PHR1*, *WRKY75* and *BHLH32*), phospholipid metabolism (*MGD2*, *SQD2*), metal binding (*LPR1*), and C-compound and carbohydrate metabolism (*PYK10*) (Figure 4 and Table 4). Among the twelve genes, *SQD2* and *GPT2* were associated with LP-specific QTL region between 72.8–87.3 cM on A10 regulating SW-LP and 50.2–57.2 cM on C1 regulating SY-LP, respectively. *PHR1*, *BHLH32* and *PHO1* were associated with the OP-specific QTL region between 43.6–48.8 cM on A1, 83.1–102.8 cM on A6 and 56.9–64.5 cM on C1 that regulate FBH-OP, BN-OP and SY-OP and SN-OP, respectively. *PHT1* and *PYK10* were linked to the constitutive QTL region between 0.0–8.4 cM on C6 regulating SN-LP/OP and 70.2–77.0 cM on A3 controling SW-LP/OP, respectively. *GPT1* and *MGD2,* and *SIZ1* were repeatedly associated with the constitutive QTL regions regulating SY-LP mapped at 77.1–91.3 cM and 91.3–95.0 cM on A2, respectively.

## Discussion

The purpose of this study was to detect P efficiency related QTLs regulating seed yield and yield-related traits using the TN DH population of *B. napus* derived from a cross between P-inefficient *cv.* Tapidor and P-efficient *cv*. Ningyou 7, and to associate QTLs with functional genes involved in P homeostasis.

### Effect of P deficiency on seed yield and yield-related traits

Both parents, Tapidor and Ningyou 7, showed significant differences in the tested traits under both low P (LP) and optimal P (OP) conditions (Figure 1). Ningyou 7 had higher number of primary branches (BN), seed weight of 1,000 seeds (SW), seed number per pod (SN) and seed yield (SY) and lower height to the first primary branch (FBH), relative first primary branch height (RBH) and pod number per plant (PN) than Tapidor under both P conditions. QTL mapping revealed that majority of the Ningyou 7 alleles among the identified QTLs were associated with increased BN, SW, SN and SY but decreased PN under both LP and OP conditions (Table 4), indicating that the contribution of Ningyou 7 to seed yield was higher than Tapidor under both P conditions.

P deficiency significantly decreased PN, BN, SN and plant height (PH), increased FBH and RBH, and had no effect on SW in the double haploid mapping population (TN DH) derived from a cross between Ningyou 7 and Tapidor (Tables 1 and 2) consistent with previous studies [19], [25] indicating that SW was genetically more tightly controlled and less influenced by environmental factors than other yield-related traits. Of the seven yield-related traits, PN, BN and RBH were most severely affected by P-deficiency (Table 1) in addition to having high correlation with SY (Table 3). Therefore, higher levels of PN and BN, and lower levels of RBH should be highly considered in the breeding of *B. napus* cultivars tolerant of environments with low P.

### Dissection of stable QTLs associated with seed yield and yield-related traits conferring P-deficiency tolerance

In this study, a total of 155 putative QTLs associated with seed yield and seven yield-related traits were identified under LP and OP conditions in all three field trials (Table 4). These QTLs were clustered and integrated into 29 LP-specific, 29 OP-specific and 23 constitutive QTL regions (Figure 4 and Table S2). The genetic architecture of seed yield in *B. napus* was previously analyzed by QTL mapping of seed yield-related traits [25], [31]–[35]. About 85 QTLs associated with seed yield were identified along with 785 QTLs for eight yield-associated traits using TN DH population from 10 natural environments with optimal P [25]. In the present study, a majority of these OP-specific QTLs were identified in only one of the three trials and 10 OP-specific QTLs, i.e. all QTLs for PH-OP mapped on A9, were located in the QTL associated with traits detected in semi-winter environments by Shi et al. [25]. These results indicated that quantitative traits were influenced both by heredity and growth conditions.

In recent studies on *B. napus* LP-specific QTLs were reported for yield-related traits [19], root morphological traits [20] and shoot traits at the vegetative stage [21]. Further analysis of these QTLs suggested that the presence of specific genes or differential expression of some genes regulated the response to P deficiency. In this study, nine QTL regions were detected for the same traits under LP conditions in at least two field trials, including three LP-specific regions and six constitutive QTL regions (Figure 4; Table S2). These stable QTL regions associated with the response to P deficiency have been compared with QTLs for P efficiency identified in a BE RIL population by comparative mapping [19]–[21], [23]. Of the three reproducible LP-specific QTL regions regulating SN-LP (56.4–66.4 cM on A2), PN-LP (80.0–92.1 cM on A3) and SW-LP (0.0–28.2 cM on A5) (Figure 4 and Table S2), the regions for SN-LP and PN-LP were co-located with the QTLs for SN and PN identified in the BE RIL population exposed to LP, respectively [19].

Among the six reproducible constitutive QTLs under LP condition, SY-LP was mapped between 77.1–91.3 cM and 91.3–95.0 cM on A2, FBH-LP between 46.0–50.8 cM and 51.6–61.5 cM on C6, PH-LP between 50.2–59.9 cM on A3 and SW-LP between 70.2–77.0 cM on A3 (Figure 4 and Table S2). The constitutive region from 77.1–91.3 cM on A2 played a major role in controlling SY-LP in Tri.1 and Tri.2 explaining 10.8–13.0% of SY-LP variation and also was detected for SN-OP in Tri.1 and Tri.3 accounting for 7.2–12.0% of SN-OP variation. The region from 91.3–95.0 cM on A2 was another reproducible locus for SY-LP in Tri.2 and Tri.3 explaining 8.4–14.2% of SY-LP variation and also played a major role in regulating SN-OP in Tri.1 and Tri.2 explaining 12.0–16.9% of SN-OP variation. This region also coincided with QTLs for SN and PN in BE population exposed to LP [19]. The two major loci associated with FBH-LP that were repeatedly detected in all three trials was located on C6 between 46.0–50.8 and 51.6–61.5 cM, accounting for 9.2–17.8% and 12.4–18.5% of FBH-LP variation, respectively. The stable region for FBH-LP (51.6–61.5 cM on C6) associated with PH-LP and PH-OP was co-located with the reproducible QTL for PH in BE RIL population exposed to LP. The reproducible locus for SW-LP located between 70.2 and 77.0 cM on A3 also coincided with the stable loci for SW in BE RIL population exposed to LP [19]. These results indicated that common mechanisms may be involved in the response of different *B. napus* cultivars to P deficiency. In addition, stable QTL regions, 50.2–59.9 cM on A3 regulating PH-LP and 46.0–50.8 cM on C6 associated with FBH-LP, co-localized with QTL regions for root morphology traits conferring P-efficiency in BE RIL population [20], [21]. This indicated that selection of early vigor plants with more developed root system at the seedling stage could enhance P acquisition, improve seed yield-related traits and therefore increase the seed yield at low P. Detection of these reproducible loci in different populations that respond to P deficiency would provide useful information to conduct marker-assisted selection to breed *B. napus* with improved P-efficiency.

### Gene prediction in *Brassica* by *in silico* comparative mapping

Unlike anonymous molecular markers which were widely used to construct *B. napus* genetic maps such as SSR and AFLP [24], [36], GBMs developed from specific genes that are closely related to phenotypic traits could be useful in associating target traits with function [37], [38]. For example, Yang *et al.* [20] found that the two mapped GBMs, BnIPS2 and BnGPT1, were linked to the QTL controlling root morphology in response to P starvation. The RI lines with favorable alleles related to BnIPS2 and BnGPT1 could develop larger root system, produce higher dry weight, acquire more P under LP conditions and produce more seeds [19], [20]. In this study, a total of 30 GBMs derived from 19 genes involved in P homeostasis in *Arabidopsis* were found to be linked to 24 QTL regions associated with yield traits (Figure 4 and Table 4). BnWRKY75-A3 and BnPHT3-A3 designed from the phosphate-starvation-response genes *AtWRKY75* and *AtPHT3;1* respectively, were associated with the constitutive QTL region regulating FBH in LP and OP conditions, and RBH and SW in OP condition and located between 12.2–18.6 cM on A3 (Table 4). They are also associated with primary root length (PRL) in LP and OP conditions, and total root length (TRL) in OP condition (data not shown). The association between functional genes and QTLs may accelerate identification of candidate genes regulating target traits.

Comparative genome analysis is commonly performed between *Brassica* species and *Arabidopsis* genome to align conserved genomic blocks and associate genes with QTLs identified in *Brassica species* [24], [39]–[41]. For example, 40 synteny blocks and 84 islands were aligned between *A. thaliana* pseudochromosomes and the TN linkage groups and 153 orthologs of the flowering time genes in *Arabidopsis* were mapped in the QTL regions by *in silico* mapping [24]. QTL clusters on chromosome C3 associated with root morphology under P starvation of *B. napus* and QTLs for shoot dry weight, shoot P content and P use efficiency under LP conditions in *B. oleracea* were found to be located in the same block which also has a number of orthologous genes for root development, auxin transport and P metabolism in *Arabidopsis* [20], [29]. However, many chromosomal regions from the present linkage maps could not be aligned with the *Arabidopsis* genome because of limited number of markers with known sequence information. The sequences of *B. napus*, *B. rapa* and *B. oleracea* are highly conserved. The annotated *B. rapa* [26] and available *B. oleracea* genome sequences (http://www.ocri-genomics.org/) along with the single nucleotide polymorphism linkage maps of *B. napus* [42], [43] will greatly facilitate the genomic alignment of *Brassica* species and allow candidate gene prediction in *B. napus*. In this study, a comparative genetic mapping was performed for the first time between the TN linkage map and the *B. rapa* and *B. oleracea* genomes. Most chromosomal regions in TN linkage groups can be aligned linearly with the *B. rapa* and *B. oleracea* genomes (Figure 4), which provided useful sequence information to develop molecular markers for QTL fine mapping in the future. Of the 19 P homeostasis genes in *Arabidopsis* located in the QTL intervals determined in this study, twelve were refracted to the corresponding segments of *B. rapa* or *B. oleracea* genomes (Figure 4 and Table 4). Among these genes, *GPT1*, *MGD2* and *SIZ1* were associated with two major loci for SY-LP, which co-located with yield-related traits between 77.1–95.0 cM on A2. *GPT1* functioning as an importer of glucose-6-phosphate in *Arabidopsis* [44] was associated with PN detected under LP conditions by Ding et al. [19]. *MGD2* encoded a type-B monogalactosyldiacylglycerol synthase involved in phospholipid metabolism and was induced under phosphate (Pi) deprivation [45]. *AtSIZ1* encoded a plant small ubiquitin-like modifier (SUMO) E3 ligase and was a focal controller of Pi starvation dependent responses [46]. These three genes would be the most likely candidates for target QTLs that confer P deficiency tolerance and near-isogenic lines were developed to confirm the function of these genes. With deep sequencing already performed on the parents of TN DH population, sequence of orthologous genes in *B. rapa* and *B. olereacea* will provide useful information in the assembly and dissection of alleles between the parents, Tapidor and Ningyou 7.

## Materials and Methods

### Plant materials and Field trials

A *B. napus* DH population with 188 lines was used in this study. The DH population was derived from a cross between Tapidor and Ningyou 7 by microspore culture [47]. Ningyou 7 was characterized as a P-efficient cultivar with better growth and higher P acquisition than Tapidor under low P (LP) and optimal P (OP) conditions in pot culture [48].

Three field trials were conducted at an experimental agricultural research station in Qichun, Hubei Province in China (115°45′N latitude, 30°19′E longitude) which has sandy paddy soil. The first trial coded Tri.1 was from Sept. 2008 to May 2009, the second trial coded Tri.2 was from Sept. 2009 to May 2010 and the third trial coded Tri.3 was from Sept. 2010 to May 2011. Soil properties were as follows: pH (1∶1 H_2_O) 4.8, organic matter 34.9 g kg^−1^, total nitrogen 0.22 g kg^−1^, available nitrogen 74 mg kg^−1^, Olsen-P 3.32 mg kg^−1^, available potassium 42 mg kg^−1^ and HWSB (hot water soluble boron) 0.09 mg kg^−1^.

The basal fertilizers included 60% N (urea, N 46%), 100% P_2_O_5_ (calcium superphosphate, P_2_O_5_ 12%), 100% K_2_O (potassium chloride, K_2_O 60%), 100% ZnSO_4_·7H_2_O and 100% Borax (Na_2_B_4_O_7_·10H_2_O) according to the following nutrient rates: N 120 kg ha^−1^, P_2_O_5_ 9 (LP) or 90 (OP) kg ha^−1^, K_2_O 150 kg ha^−1^, ZnSO_4_·7H_2_O 45 kg ha^−1^ and Borax (Na_2_B_4_O_7_·10H_2_O) 15 kg ha^−1^. Rest of the urea was applied before the green bud stage. 90 and 9 kg ha^−1^ of P_2_O_5_ (calcium superphosphate, P_2_O_5_ 12%) were applied to create OP and LP conditions before transplantation. Three replications for 188 TN DH lines and their parents were planted in a randomized plot design with each plot comprising 18 plants, separated by a distance of 0.20 m between plants and 0.28 m between rows.

Seeds were sown in a nursery bed in the field in middle September and seedlings were transplanted 30 d after sowing. Plants were harvested in the following middle May. Standard agricultural practices were followed for field management.

### Measurement of phenotypic traits

In each plot, six individuals from the middle row were used to determine height to the first primary branch (FBH) measured from ground level to the base of the lowest primary branch, plant height (PH) measured from ground level to the tip of the main inflorescence, number of primary branches (BN) measured as the number of primary branches arising from main shoot and seed number per pod (SN) measured as the average number of well-filled seeds from 100 well-developed pods sampled from the primary branch in the middle of each plant studied. All representative individuals from each plot were harvested by hand at maturity stage to investigate seed yield per plant (SY) and seed weight of 1,000 seeds (SW). Pod number per plant (PN) was calculated using the following formula: PN = SY×1000/(SW×SN). Relative first primary branch height (RBH) was defined as the ratio of FBH to PH in each sampled plant. Tested traits were denoted with ‘trait name and treatment name’ such as PH-LP which indicates plant height under low P conditions.

### Linkage map construction and alignment with *B. rapa* and *B. oleracea* genomes

About 53 GBMs designed from 46 genes regulating P homeostasis in *Arabidopsis* [49] were superimposed on the framework of the existing well-defined G-map [50]. In total, the new genetic linkage map had 798 molecular markers with the average distance of 2.57 cM between adjacent markers. Linkage map was constructed using JoinMap4.0 [51] and mapping was performed according to the method described by Long et al. [50]. Order of markers on the new linkage map was consistent with previously published maps [25], [50].

The physical location of markers along with primer sequences in the TN DH linkage map present on *B. rapa* or *B. oleracea* genome was determined by *in silico* PCR. Primer sequences of markers were first aligned with *B. rapa* and *B. oleacea* genome sequences using BlastN with default parameters on the web site (http://brassicadb.org/, http://www.ocri-genomics.org/). Physical position of the primer alignment on *B. rapa* or *B. oleracea* genome was determined by matching primer pairs that amplified a 200∼2000 bp fragment. Physical position of markers was determined by aligning the product sequence of the TN DH linkage map against the *B. rapa* and *B. oleracea* genomes with e-value ≤1e^−10^ and at least 50% of the sequence length covered. Position of these markers on *B. rapa* and *B. oleracea* genomes were integrated and sorted according to their position on corresponding chromosomes and then were compared with the TN DH linkage groups for map alignment. For markers with multiple alignment positions, the best position was determined based on neighboring markers on the same linkage group.

### Statistical analysis and QTL detection

Data analysis was conducted using SAS 8.1 (SAS Institute, Cary, NC, USA). Fisher's least significant difference (LSD) was used to test the significance of means at 0.05 levels. Correlation analysis was conducted to determine the relationship between the tested traits.

QTLs were detected by composite interval mapping (CIM) using WinQTL cartographer 2.5 software (http://statgen.ncsu.edu/qtlcar/WQTLCart.htm; [52]). For each trait, QTL threshold (*P*<0.05) was estimated from 1,000 permutations [53]. Each QTL was denominated as “*q*” (abbreviation of QTL)+trait name+trial number+chromosome name+the serial letter. For example, *qBN-LP1-A1a* and *qBN-LP1-A1b* denote two QTLs for number of primary branches (BN) detected on chromosome A1 under LP condition in Tri. 1.

## Supporting Information

Table S1 **Seed yield and yield-related traits in 188 lines of a double haploid mapping population (TN DH) derived from a cross between a P-efficient cultivar, Ningyou 7 and a P-inefficient cultivar, Tapidor under low (LP) and optimal phosphorus (OP) conditions in three field trials.**
(XLSX)

Table S2 **Quantitative trait loci (QTLs) associated with seed yield and yield-related traits under low (LP) and optimal phosphorus (OP) conditions and distribution of gene-based markers (GBM) in the QTL intervals.**
(DOC)
