# Supplementary material for: QTL for Yield Traits and Their Association with Functional Genes in Response to Phosphorus Deficiency in Brassica napus
Source: PLoS One. 2013 Jan 28;8(1):e54559. doi: 10.1371/journal.pone.0054559 (PMC3557265; doi:10.1371/journal.pone.0054559)
Supplement: Table S2 — Quantitative trait loci (QTLs) associated with seed yield and yield-related traits under low (LP) and optimal phosphorus (OP) conditions and distribution of gene-based markers (GBM) in the QTL intervals. (DOC) [file pone.0054559.s002.doc]

**Table S2 Quantitative trait loci (QTLs) associated with seed yield and yield-related traits under low P (LP) and optimal P (OP) conditions and distribution of gene-based markers (GBM) in the QTL** intervals

| P level | Linkage | Region | Trait | QTL | LOD | R^2a^ | Position | CI^b^ | AE^c^ | GBM^d^ | Gene name^e^ |
| --- | --- | --- | --- | --- | --- | --- | --- | --- | --- | --- | --- |
| LP | A1 | 19.6−28.9 | FBH-LP | *qFBH-LP1-A1a* | 4.5 | 7.6 | 24.8 | 19.6−28.9 | + |  |  |
| LP | A1 | 74.0−82.5 | BN-LP | *qBN-LP1-A1a* | 5.3 | 11.0 | 77.9 | 74.0−82.5 | - | BnPAP17-A1 | *AtPAP17* |
| LP | A1 | 82.5−96.7 | BN-LP | *qBN-LP1-A1b* | 5.5 | 12.3 | 88.7 | 82.5−96.7 | - |  |  |
| LP | A2 | 7.9−12.1 | BN-LP | *qBN-LP2-A2a* | 4.4 | 9.3 | 8.5 | 7.9−12.1 | - |  |  |
| LP | A2 | 56.4−66.4 | SN-LP | *qSN-LP1-A2a* | 4.5 | 7.9 | 57.8 | 56.4−58.9 | - |  |  |
|  |  |  | SN-LP | *qSN-LP1-A2b* | 3.7 | 6.5 | 62.8 | 61.2−65.7 | - |  |  |
|  |  |  | SN-LP | *qSN-LP2-A2* | 3.6 | 6.8 | 62.8 | 57.8−66.4 | - |  |  |
| LP | A3 | 27.8−32.3 | PN-LP | *qPN-LP2-A3a* | 3.4 | 6.6 | 28.6 | 27.8−32.3 | + |  |  |
|  |  |  | SW-LP | *qSW-LP1-A3c* | 4.7 | 8.8 | 30.6 | 28.6−31.9 | - |  |  |
| LP | A3 | 46.0−49.4 | PH-LP | *qPH-LP2-A3a* | 4.8 | 8.7 | 47.5 | 46.0−49.4 | + |  |  |
| LP | A3 | 58.5−61.5 | SN-LP | *qSN-LP3-A3* | 4.2 | 8.0 | 59.5 | 58.5−61.5 | + |  |  |
|  |  |  | PH-LP | *qPH-LP2-A3c* | 4.1 | 7.4 | 60.3 | 59.5-61.5 | + |  |  |
| LP | A3 | 80.0−92.1 | SW-LP | *qSW-LP1-A3e* | 5.6 | 9.7 | 82.9 | 80.0−88.7 | - |  |  |
|  |  |  | PN-LP | *qPN-LP1-A3* | 3.6 | 7.9 | 87.0 | 82.3−90.7 | + |  |  |
|  |  |  | PN-LP | *qPN-LP2-A3c* | 3.4 | 6.5 | 87.0 | 81.5−89.4 | + |  |  |
|  |  |  | SY-LP | *qSY-LP2-A3* | 4.8 | 8.8 | 87.0 | 82.2−92.1 | + |  |  |
| LP | A4 | 57.5−84.9 | RBH-LP | *qRBH-LP1-A4* | 3.6 | 6.7 | 68.7 | 57.5−72.2 | - | BnLPR2-A4 | *AtLPR2* |
|  |  |  | PN-LP | *qPN-LP3-A4* | 3.5 | 7.5 | 68.9 | 65.4−73.5 | + | BnPHF1-A4 | *AtPHF1* |
|  |  |  | BN-LP | *qBN-LP1-A4* | 3.4 | 6.5 | 73.5 | 69.9−84.9 | + |  |  |
| LP | A5 | 0.0−18.3 | SW-LP | *qSW-LP3-A5* | 3.8 | 6.6 | 14.4 | 0.0−18.3 | - |  |  |
| LP | A5 | 16.3−28.2 | FBH-LP | *qFBH-LP2-A5* | 3.6 | 6.6 | 19.3 | 16.3−22.8 | + |  |  |
|  |  |  | SW-LP | *qSW-LP1-A5a* | 3.9 | 6.1 | 19.9 | 19.3−28.2 | - |  |  |
|  |  |  | SW-LP | *qSW-LP2-A5a* | 3.6 | 6.7 | 22.8 | 19.6−28.2 | - |  |  |
| LP | A5 | 30.9−37.7 | SW-LP | *qSW-LP1-A5b* | 4.0 | 6.7 | 32.8 | 28.2−36.3 | - | BnPHT1-A5 | *AtPHT1* |
| LP | A5 | 56.8−59.1 | BN-LP | *qBN-LP3-A5* | 3.7 | 8.9 | 57.8 | 56.8−59.1 | + |  |  |
| LP | A7 | 36.3−47.6 | RBH-LP | *qRBH-LP2-A7a* | 4.0 | 7.9 | 42.6 | 36.3−47.6 | - |  |  |
| LP | A9 | 10.6−16.4 | PN-LP | *qPN-LP1-A9* | 4.2 | 9.1 | 12.6 | 10.6−16.4 | + |  |  |
| LP | A9 | 16.4−21.0 | PN-LP | *qPN-LP2-A9* | 4.9 | 9.5 | 17.4 | 16.4−21.0 | + |  |  |
| LP | A9 | 66.0−71.5 | PH-LP | *qPH-LP3-A9a* | 4.4 | 9.4 | 68.6 | 66.0−71.5 | - | BnLPR1-A9c/d/e | *AtLPR1* |
| LP | A9 | 76.2−82.7 | PH-LP | *qPH-LP3-A9b* | 4.7 | 10.0 | 77.7 | 76.2−82.7 | - |  |  |
| LP | A9 | 124.4−131.7 | SN-LP | *qSN-LP1-A9b* | 4.7 | 8.4 | 126.5 | 124.4−131.7 | + |  |  |
| LP | A10 | 64.0−72.8 | SW-LP | *qSW-LP3-A10a* | 3.7 | 7.9 | 67.1 | 64.0−72.8 | - |  |  |
| LP | A10 | 72.8−87.3 | SW-LP | *qSW-LP3-A10b* | 3.8 | 7.4 | 76.6 | 72.8−87.3 | - | BnSQD2-A10a/c | *AtSQD2* |
| LP | C1 | 40.8−48.6 | SY-LP | *qSY-LP1-C1a* | 3.5 | 6.4 | 46.1 | 40.8−46.5 | - |  |  |
|  |  |  | SN-LP | *qSN-LP2-C1* | 3.6 | 7.1 | 47.1 | 46.5−48.6 | - |  |  |
| LP | C1 | 50.2−57.2 | SY-LP | *qSY-LP1-C1b* | 3.5 | 7.4 | 54.2 | 50.2−57.2 | - | BnGPT2-C1 | *AtGPT2* |
| LP | C3 | 52.6−58.8 | PN-LP | *qPN-LP3-C3a* | 3.4 | 7.4 | 57.5 | 52.6−58.8 | - |  |  |
| LP | C3 | 68.6−71.9 | PN-LP | *qPN-LP3-C3b* | 5.5 | 12.3 | 69.0 | 68.6−71.9 | + |  |  |
| LP | C6 | 61.5−68.5 | FBH-LP | *qFBH-LP2-C6c* | 6.2 | 12.9 | 63.5 | 61.5−68.5 | - |  |  |
| LP | C7 | 19.1−38.5 | FBH-LP | *qFBH-LP1-C7* | 3.5 | 5.7 | 29.8 | 19.1−38.5 | + | BnRNS1-C7 | *AtRNS1* |
| LP | C9 | 61.9−84.8 | FBH-LP | *qFBH-LP3-C9* | 4.3 | 8.2 | 75.1 | 61.9−84.8 | - |  |  |
|  |  |  |  |  |  |  |  |  |  |  |  |
| LP&OP | A1 | 30.1−38.2 | FBH-LP | *qFBH-LP1-A1b* | 5.8 | 9.6 | 33.1 | 32.5−36.7 | + | BnPHR1-A1a | *AtPHR1* |
|  |  |  | RBH-OP | *qRBH-OP2-A1* | 5.2 | 10.7 | 34.1 | 33.1−37.9 | + |  |  |
|  |  |  | FBH-OP | *qFBH-OP2-A1a* | 5.1 | 8.8 | 35.6 | 34.9−37.6 | + |  |  |
|  |  |  | RBH-LP | *qRBH-LP3-A1* | 3.6 | 9.3 | 36.6 | 30.1−38.2 | + |  |  |
| LP&OP | A2 | 20.0−35.1 | BN-LP | *qBN-LP2-A2b* | 3.3 | 8.6 | 23.4 | 20.0−31.8 | + |  |  |
|  |  |  | PH-LP | *qPH-LP3-A2* | 6.0 | 14.3 | 25.4 | 22.2−35.1 | - |  |  |
|  |  |  | PH-OP | *qPH-OP1-A2a* | 5.0 | 8.4 | 26.0 | 21.1−29.0 | - |  |  |
| LP&OP | A2 | 77.1−91.3 | SY-LP | *qSY-LP1-A2a* | 5.9 | 12.1 | 80.0 | 77.1−81.8 | - |  |  |
|  |  |  | SY-OP | *qSY-OP1-A2* | 4.6 | 9.2 | 80.3 | 78.0−83.5 | - |  |  |
|  |  |  | SY-LP | *qSY-LP2-A2a* | 5.8 | 13.0 | 84.5 | 84.0−86.5 | - |  |  |
|  |  |  | SN-OP | *qSN-OP3-A2* | 3.6 | 7.4 | 83.5 | 79.3−86.5 | - |  |  |
|  |  |  | SN-OP | *qSN-OP1-A2a* | 5.9 | 12.0 | 84.5 | 83.3−87.0 | - |  |  |
|  |  |  | SN-LP | *qSN-LP3-A2* | 3.9 | 8.7 | 84.5 | 83.0−91.0 | - |  |  |
|  |  |  | RBH-LP | *qRBH-LP2-A2* | 3.7 | 7.5 | 83.5 | 79.0−87.0 | + |  |  |
|  |  |  | SW-OP | *qSW-OP3-A2* | 3.6 | 7.1 | 86.5 | 85.7−91.3 | - | BnGPT1-A2 | *AtGPT1* |
|  |  |  | SY-LP | *qSY-LP1-A2b* | 4.8 | 10.8 | 89.0 | 87.0−91.0 | - | BnSIZ1-A2 | *AtSIZ1* |
| LP&OP | A2 | 91.3−95.0 | RBH-OP | *qRBH-OP3-A2* | 9.5 | 20.0 | 93.3 | 91.8−94.0 | + |  |  |
|  |  |  | SN-OP | *qSN-OP1-A2b* | 6.8 | 12.0 | 94.0 | 91.7−95.0 | - | BnMGD2-A2a/b | *AtMGD2* |
|  |  |  | SN-OP | *qSN-OP2-A2* | 9.0 | 16.9 | 94.0 | 92.4-95.0 | - |  |  |
|  |  |  | SY-LP | *qSY-LP2-A2b* | 7.4 | 14.2 | 94.0 | 93.1−95.0 | - |  |  |
|  |  |  | SY-LP | *qSY-LP3-A2* | 4.1 | 8.4 | 94.0 | 91.3−95.0 | - |  |  |
| LP&OP | A3 | 5.7−12.2 | FBH-OP | *qFBH-OP3-A3b* | 4.4 | 9.8 | 10.0 | 5.7−12.0 | + |  |  |
|  |  |  | SW-LP | *qSW-LP1-A3a* | 3.6 | 7.0 | 10.0 | 8.8−12.2 | - |  |  |
| LP&OP | A3 | 12.2−18.6 | FBH-OP | *qFBH-OP1-A3* | 3.5 | 6.4 | 14.2 | 12.2−18.3 | + | BnWRKY75-A3 | *AtWRKY75* |
|  |  |  | RBH-OP | *qRBH-OP1-A3* | 3.6 | 6.9 | 14.2 | 12.2−17.8 | + | BnPHT3-A3 | *AtPHT3* |
|  |  |  | FBH-LP | *qFBH-LP1-A3* | 3.8 | 6.2 | 15.5 | 12.2−18.6 | + |  |  |
|  |  |  | SW-OP | *qSW-OP2-A3a* | 4.7 | 7.9 | 15.5 | 14.4−17.0 | - |  |
| LP&OP | A3 | 17.5−27.8 | SW-LP | *qSW-LP1-A3b* | 6.9 | 12.0 | 21.9 | 17.5−22.8 | - |  |  |
|  |  |  | SW-OP | *qSW-OP2-A3b* | 3.7 | 6.5 | 22.8 | 20.9−27.8 | - |  |  |
| LP&OP | A3 | 38.9−44.2 | FBH-OP | *qFBH-OP2-A3b* | 6.2 | 10.0 | 39.4 | 38.9−40.4 | + |  |  |
|  |  |  | PN-LP | *qPN-LP2-A3b* | 4.5 | 8.6 | 40.8 | 39.4−44.2 | + |  |  |
| LP&OP | A3 | 50.2−59.9 | PH-LP | *qPH-LP1-A3a* | 4.4 | 7.9 | 51.9 | 50.2−52.9 | + |  |  |
|  |  |  | PH-LP | *qPH-LP2-A3b* | 4.9 | 9.7 | 54.9 | 51.2−59.9 | + |  |  |
|  |  |  | PH-LP | *qPH-LP1-A3b* | 5.4 | 9.2 | 58.5 | 52.9−59.8 | + |  |  |
|  |  |  | SW-OP | *qSW-OP1-A3a* | 8.9 | 17.7 | 55.9 | 52.3−58.5 | - |  |  |
| LP&OP | A3 | 70.2−77.0 | SW-LP | *qSW-LP1-A3d* | 5.9 | 11.5 | 70.7 | 70.2−74.6 | - | BnPYK10-A3a/b | *AtPYK10* |
|  |  |  | SW-LP | *qSW-LP3-A3* | 4.4 | 7.8 | 73.7 | 70.5−77.0 | - |  |  |
|  |  |  | SW-OP | *qSW-OP1-A3c* | 10.7 | 20.2 | 72.7 | 70.2−76.5 | - |  |  |
| LP&OP | A3 | 87.0−94.0 | PN-OP | *qPN-OP1-A3a* | 3.7 | 7.4 | 89.0 | 87.0−92.1 | + |  |  |
|  |  |  | SW-LP | *qSW-LP1-A3f* | 4.1 | 6.7 | 91.7 | 89.4−94.0 | - |  |  |
| LP&OP | A3 | 114.0−127.3 | PN-OP | *qPN-OP2-A3* | 3.7 | 7.8 | 116.3 | 114−122.8 | + | BnSQD1-A3 | *AtSQD1* |
|  |  |  | FBH-LP | *qFBH-LP2-A3* | 3.5 | 6.3 | 121.3 | 114.7−127.3 | + |  |  |
| LP&OP | A4 | 8.6−21.8 | SW-LP | *qSW-LP1-A4* | 6.0 | 9.6 | 18.7 | 17.4−21.8 | - |  |  |
|  |  |  | SW-OP | *qSW-OP1-A4a* | 4.3 | 6.8 | 19.3 | 8.6−21.4 | - |  |  |
| LP&OP | A5 | 36.3−49.4 | SW-LP | *qSW-LP2-A5b* | 4.5 | 8.4 | 37.7 | 36.3-48.6 | - |  |  |
|  |  |  | SW-OP | *qSW-OP2-A5* | 4.7 | 8.3 | 37.7 | 36.5−44.5 | - |  |  |
|  |  |  | PN-OP | *qPN-OP1-A5* | 3.4 | 6.6 | 40.0 | 38.8−49.4 | + |  |  |
| LP&OP | A7 | 43.1−54.3 | SW-OP | *qSW-OP2-A7a* | 6.2 | 11.1 | 47.6 | 43.1−54.3 | - |  |  |
|  |  |  | RBH-LP | *qRBH-LP2-A7b* | 3.4 | 7.0 | 50.6 | 48.0−54.3 | - |  |  |
| LP&OP | A9 | 115.4−122.6 | SN-LP | *qSN-LP1-A9a* | 4.2 | 7.4 | 119.8 | 115.4−120.9 | + |  |  |
|  |  |  | SN-OP | *qSN-OP2-A9* | 3.6 | 6.3 | 119.8 | 115.6−122.6 | + |  |  |
| LP&OP | C6 | 0.0−8.4 | SN-LP | *qSN-LP3-C6* | 4.0 | 8.6 | 1.0 | 0.0−7.7 | + | BnPHT1-C6 | *AtPHT1* |
|  |  |  | SN-OP | *qSN-OP3-C6a* | 3.9 | 9.8 | 4.0 | 0.0−8.4 | + |  |  |
| LP&OP | C6 | 8.4−25.4 | SN-OP | *qSN-OP3-C6b* | 4.0 | 9.0 | 12.4 | 8.4−21.7 | + |  |  |
|  |  |  | SN-LP | *qSN-LP1-C6* | 4.3 | 8.2 | 16.4 | 10.6−25.4 | + |  |  |
| LP&OP | C6 | 46.0−50.8 | FBH-LP | *qFBH-LP1-C6a* | 5.3 | 9.2 | 46.4 | 46.0−47.2 | - |  |  |
|  |  |  | FBH-LP | *qFBH-LP2-C6a* | 6.1 | 11.1 | 46.4 | 46.0−46.6 | - |  |  |
|  |  |  | FBH-LP | *qFBH-LP3-C6a* | 8.7 | 17.8 | 47.6 | 47.2−50.8 | - |  |  |
|  |  |  | FBH-OP | *qFBH-OP2-C6a* | 6.0 | 9.9 | 47.8 | 46.6−50.8 | - |  |  |
| LP&OP | C6 | 51.6−61.5 | FBH-OP | *qFBH-OP3-C6b* | 4.2 | 8.0 | 55.6 | 51.6−57.0 | - |  |  |
|  |  |  | FBH-LP | *qFBH-LP3-C6b* | 9.1 | 18.5 | 56.4 | 52.9−60.8 | - |  |  |
|  |  |  | FBH-LP | *qFBH-LP1-C6b* | 7.0 | 12.4 | 57.4 | 56.4−61.5 | - |  |  |
|  |  |  | FBH-LP | *qFBH-LP2-C6b* | 7.2 | 13.4 | 57.4 | 52.7−61.5 | - |  |  |
|  |  |  | PH-LP | *qPH-LP2-C6* | 4.5 | 8.8 | 57.4 | 54.0−61.5 | - |  |  |
|  |  |  | PH-OP | *qPH-OP2-C6* | 3.5 | 6.8 | 57.4 | 51.6−61.5 | - |  |  |
|  |  |  | FBH-OP | *qFBH-OP2-C6b* | 7.8 | 13.8 | 59.4 | 53.8−61.5 | - |  |  |
| LP&OP | C7 | 40.0−51.5 | SY-LP | *qSY-LP3-C7* | 3.6 | 7.3 | 47.6 | 40.0−50.6 | - |  |  |
|  |  |  | PH-OP | *qPH-OP1-C7a* | 3.6 | 6.6 | 48.6 | 47.1−51.5 | + |  |  |
| LP&OP | C9 | 7.5−31.5 | FBH-LP | *qFBH-LP1-C9* | 4.0 | 6.5 | 18.0 | 12.6−29.6 | - |  |  |
|  |  |  | RBH-OP | *qRBH-OP2-C9* | 3.4 | 6.6 | 18.0 | 7.5−31.5 | - |  |  |
| LP&OP | C9 | 86.7−105.1 | SY-OP | *qSY-OP3-C9* | 4.8 | 10.2 | 88.1 | 86.7−89.4 | - |  |  |
|  |  |  | SY-OP | *qSY-OP1-C9* | 3.5 | 7.7 | 93.4 | 87.1-105.1 | - |  |  |
|  |  |  | PN-OP | *qPN-OP3-C9* | 4.3 | 10.0 | 93.4 | 86.9−105.1 | - |  |  |
|  |  |  | BN-LP | *qBN-LP2-C9* | 4.4 | 10.3 | 100.1 | 88.3−105.1 | - |  |  |
|  |  |  |  |  |  |  |  |  |  |  |  |
| OP | A1 | 43.6−48.8 | FBH-OP | *qFBH-OP2-A1b* | 7.7 | 12.7 | 46.7 | 43.6−48.8 | - | BnPHR1-A1b | *AtPHR1* |
| OP | A2 | 35.2−37.5 | PH-OP | *qPH-OP1-A2b* | 3.4 | 5.9 | 37.2 | 35.2−37.5 | - |  |  |
| OP | A3 | 0.0−5.7 | FBH-OP | *qFBH-OP3-A3a* | 4.4 | 8.0 | 0.0 | 0.0−5.7 | - |  |  |
|  |  |  | RBH-OP | *qRBH-OP3-A3* | 3.5 | 6.4 | 0.0 | 0.0−5.7 | + |  |  |
| OP | A3 | 28.4−34.8 | FBH-OP | *qFBH-OP2-A3a* | 4.5 | 8.0 | 33.3 | 28.4−34.4 | + | BnPHT1-A3 | *AtPHT1* |
|  |  |  | PH-OP | *qPH-OP3-A3* | 3.6 | 7.9 | 33.6 | 32.6−34.8 | + |  |  |
| OP | A3 | 36.0−39.6 | SW-OP | *qSW-OP3-A3a* | 6.7 | 12.4 | 37.5 | 36.0−39.6 | - |  |  |
| OP | A3 | 59.8−63.4 | SW-OP | *qSW-OP1-A3b* | 10.4 | 17.6 | 62.4 | 60.0−63.4 | - |  |  |
|  |  |  | SW-OP | *qSW-OP3-A3b* | 4.5 | 8.3 | 62.4 | 59.8−63.4 | - |  |  |
| OP | A3 | 92.1−98.0 | PN-OP | *qPN-OP1-A3b* | 3.8 | 8.0 | 95.7 | 92.1−98.0 | + |  |  |
| OP | A4 | 21.4−29.7 | SW-OP | *qSW-OP1-A4b* | 3.6 | 5.9 | 24.8 | 21.4−29.7 | - | BnPHT1-A4 | *AtPHT1* |
| OP | A5 | 73.5−75.6 | FBH-OP | *qFBH-OP2-A5a* | 3.8 | 6.0 | 73.6 | 73.5−75.6 | + |  |  |
| OP | A5 | 75.6−93.4 | FBH-OP | *qFBH-OP2-A5b* | 4.5 | 7.3 | 85.4 | 75.6−93.4 | + |  |  |
| OP | A6 | 83.1−102.8 | BN-OP | *qBN-OP1-A6* | 3.3 | 6.6 | 90.0 | 83.1−102.8 | - | BnBHLH32-A6 | *AtBHLH32* |
|  |  |  |  |  |  |  |  |  |  | BnPHT1-A6 | *AtPHT1* |
| OP | A7 | 14.7−18.9 | SW-OP | *qSW-OP1-A7* | 3.9 | 6.1 | 17.0 | 14.7−18.9 | - |  |  |
| OP | A7 | 55.4−62.3 | SW-OP | *qSW-OP2-A7b* | 6.2 | 11.0 | 59.0 | 55.4−62.3 | - |  |  |
| OP | A7 | 93.1−97.7 | BN-OP | *qBN-OP3-A7* | 3.7 | 7.7 | 94.1 | 93.1−97.7 | - |  |  |
| OP | A9 | 18.2−28.1 | FBH-OP | *qFBH-OP1-A9a* | 5.3 | 9.6 | 21.6 | 18.3−28.1 | + |  |  |
|  |  |  | RBH-OP | *qRBH-OP1-A9a* | 3.8 | 7.1 | 21.6 | 18.2−27.7 | - |  |  |
| OP | A9 | 28.7−31.0 | PH-OP | *qPH-OP1-A9a* | 6.6 | 11.3 | 29.1 | 28.7−29.8 | - |  |  |
|  |  |  | FBH-OP | *qFBH-OP1-A9b* | 4.7 | 8.6 | 30.3 | 28.8−31.0 | - |  |  |
| OP | A9 | 36.9−43.7 | PH-OP | *qPH-OP1-A9b* | 8.0 | 13.5 | 38.8 | 36.9−41.2 | - |  |  |
|  |  |  | PH-OP | *qPH-OP2-A9a* | 3.7 | 7.0 | 41.2 | 40.8−43.7 | - |  |  |
| OP | A9 | 47.2−53.0 | PH-OP | *qPH-OP2-A9b* | 4.6 | 8.7 | 51.1 | 47.2−53.0 | - |  |  |
| OP | A9 | 55.1−63.1 | PH-OP | *qPH-OP2-A9c* | 4.6 | 9.5 | 59.3 | 55.1−63.1 | - | BnLPR1-A9a | *AtLPR1* |
| OP | A9 | 128.2−141.2 | FBH-OP | *qFBH-OP1-A9c* | 3.8 | 7.9 | 134.2 | 128.2−141.2 | - | BnDGD2-A9 | *AtDGD2* |
|  |  |  | RBH-OP | *qRBH-OP1-A9b* | 4.4 | 10.3 | 137.2 | 128.7−141.2 | + |  |  |
| OP | C1 | 56.9−64.5 | SY-OP | *qSY-OP1-C1* | 3.8 | 8.7 | 58.5 | 56.9−62.5 | - | BnPHO1-C1 | *AtPHO1* |
|  |  |  | SN-OP | *qSN-OP2-C1* | 3.5 | 7.5 | 59.5 | 56.9−64.5 | - |  |  |
| OP | C1 | 67.6−70.8 | SN-OP | *qSN-OP1-C1* | 3.7 | 6.3 | 68.7 | 67.6−70.8 | - |  |  |
| OP | C6 | 28.9−46.4 | BN-OP | *qBN-OP2-C6* | 3.6 | 7.6 | 37.6 | 28.9−46.0 | - |  |  |
|  |  |  | FBH-OP | *qFBH-OP3-C6a* | 5.4 | 9.8 | 44.8 | 42.1−45.9 | + |  |  |
|  |  |  | RBH-OP | *qRBH-OP3-C6* | 3.7 | 6.7 | 44.8 | 41.0−46.4 | - |  |  |
| OP | C7 | 53.2−61.5 | PH-OP | *qPH-OP1-C7b* | 5.9 | 10.6 | 57.0 | 53.2−61.5 | + |  |  |
| OP | C7 | 64.1−68.7 | PH-OP | *qPH-OP1-C7c* | 3.7 | 6.2 | 67.7 | 64.1−68.7 | + |  |  |
| OP | C8 | 44.5−48.3 | SW-OP | *qSW-OP1-C8* | 4.4 | 6.8 | 47.7 | 44.5−48.3 | - |  |  |
| OP | C9 | 49.0−52.5 | FBH-OP | *qFBH-OP3-C9a* | 3.6 | 6.8 | 50.3 | 49.0−52.5 | - |  |  |
| OP | C9 | 52.5−54.1 | RBH-OP | *qRBH-OP3-C9a* | 3.5 | 7.0 | 53.0 | 52.5−54.1 | - |  |  |
| OP | C9 | 54.1−70.3 | FBH-OP | *qFBH-OP3-C9b* | 4.9 | 9.9 | 59.1 | 58.0−68.8 | - |  |  |
|  |  |  | RBH-OP | *qRBH-OP3-C9b* | 3.8 | 8.0 | 59.1 | 54.1−70.3 | - |  |  |

Note: Height to the first primary branch (cm; FBH), plant height (cm; PH), relative first primary branch height (the ratio of FBH to PH; RBH), number of primary branches per plant (N; BN), seed weight of 1,000 seeds (g per 1000 seeds; SW), seed number per pod (N; SN), pod number per plant (N; PN), seed yield per hectare (kg·ha^−1^; SY). ^a^ the explained phenotypic variation; ^b^ Confidence interval at *P* = 0.05; ^c^ Additive effects, positive value (+) means the allele came from the parent ‘Tapidor’, negative value (−) means the allele came from the parent ‘Ningyou7’; ^d^ Gene based markers designed from genes in the P metabolic pathway in *Arabidopsis* that are located in the confidence interval; ^e^ Gene name in *Arabidopsis*.
